# Targeting epigenetic and post-translational modifications of NRF2: key regulatory factors in disease treatment

**DOI:** 10.1038/s41420-025-02491-z

**Published:** 2025-04-21

**Authors:** Xinyi Yang, Yingchao Liu, Jinghao Cao, Cuiyun Wu, Lusheng Tang, Wenxia Bian, Yuhan Chen, Lingyan Yu, Yunyi Wu, Sainan Li, Yuhuan Shen, Jun Xia, Jing Du

**Affiliations:** 1 Laboratory Medicine Center, Department of Clinical Laboratory, Zhejiang Provincial People’s Hospital (Affiliated People’s Hospital), Hangzhou Medical College, Hangzhou, Zhejiang 310014 China; 2 Cancer Center, Department of Radiology, Zhejiang Provincial People’s Hospital (Affiliated People’s Hospital), Hangzhou Medical College, Hangzhou, Zhejiang China

**Keywords:** Epigenetics, Post-translational modifications

## Abstract

Nuclear factor erythroid 2-related factor 2 (NRF2) is a key transcription factor involved in regulating cellular antioxidant defense and detoxification mechanisms. It mitigates oxidative stress and xenobiotic-induced damage by inducing the expression of cytoprotective enzymes, including HO-1 and NQO1. NRF2 also modulates inflammatory responses by inhibiting pro-inflammatory genes and mediates cell death pathways, including apoptosis and ferroptosis. Targeting NRF2 offers potential therapeutic avenues for treating various diseases. NRF2 is regulated through two principal mechanisms: post-translational modifications (PTMs) and epigenetic alterations. PTMs, including phosphorylation, ubiquitination, and acetylation, play a pivotal role in modulating NRF2’s stability, activity, and subcellular localization, thereby precisely controlling its function in the antioxidant response. For instance, ubiquitination can lead to NRF2 degradation and reduced antioxidant activity, while deubiquitination enhances its stability and function. Epigenetic modifications, such as DNA methylation, histone modifications, and interactions with non-coding RNAs (e.g., MALAT1, PVT1, MIR4435-2HG, and TUG1), are essential for regulating NRF2 expression by modulating chromatin architecture and gene accessibility. This paper systematically summarizes the molecular mechanisms by which PTMs and epigenetic alterations regulate NRF2, and elucidates its critical role in cellular defense and disease. By analyzing the impact of PTMs, such as phosphorylation, ubiquitination, and acetylation, as well as DNA methylation, histone modifications, and non-coding RNA interactions on NRF2 stability, activity, and expression, the study reveals the complex cellular protection network mediated by NRF2. Furthermore, the paper explores how these regulatory mechanisms affect NRF2’s roles in oxidative stress, inflammation, and cell death, identifying novel therapeutic targets and strategies. This provides new insights into the treatment of NRF2-related diseases, such as cancer, neurodegenerative disorders, and metabolic syndrome. This research deepens our understanding of NRF2’s role in cellular homeostasis and lays the foundation for the development of NRF2-targeted therapies.

## Facts

1. The context-dependent regulation of NRF2 creates therapeutic dilemmas: While NRF2 activation shows promise for treating neurodegenerative diseases by enhancing antioxidant defenses, its inhibition may be required in cancers where NRF2 hyperactivation promotes tumor survival and drug resistance.
2. Precision targeting of PTMs remains challenging: Although specific phosphorylation sites and ubiquitination patterns are known to regulate NRF2 stability, developing modifiers that selectively target these modifications without affecting other pathways is technically demanding.
3. Epigenetic complexity in NRF2 regulation: While DNA methylation of the NRF2 promoter is well-characterized, the functional consequences of newly identified histone modifications and their crosstalk with long non-coding RNAs require further elucidation.
4. Translational gaps in therapeutic development: Current NRF2-targeting strategies struggle to balance efficacy and specificity, with small-molecule inhibitors/activators showing poor selectivity and epigenetic-based approaches facing delivery challenges in vivo.

## Questions

1. What is the ultimate molecular switch that determines whether NRF2 acts as a cytoprotective factor or a tumor promoter in different disease contexts?
2. What are the normal physiological functions of NRF2’s epigenetic regulation, particularly in maintaining cellular homeostasis under basal conditions?
3. How to precisely target NRF2 modifications in diseased cells while preserving its normal function in healthy cells, and are there cell-type specific markers that predict NRF2 modification patterns?
4. How do different PTMs of NRF2 (ubiquitination, phosphorylation, acetylation) interact and compete during cellular stress responses, and what is their hierarchical relationship in regulating NRF2 activity?

## Introduction

Within cells, NRF2 serves as a crucial transcription factor and plays an essential role in maintaining the balance of oxidative-reductive reactions and responding to oxidative stress. NRF2 enhances the cell’s capacity to manage oxidative stress and ionizing radiation, as well as other exogenous and endogenous challenges, by regulating the expression of various antioxidant genes, such as glutathione S-transferase, glutathione reductase, and NAD(P)H quinone oxidoreductase [1]. The regulatory network of NRF2 extends beyond antioxidant stress responses, encompassing a diverse range of biological processes such as cell survival and proliferation [2], metabolic balance [3], and inflammatory response [4]. This includes cellular metabolic balance, including glucose metabolism [5], lipid metabolism [6], and amino acid metabolism. Recent studies have shown that NRF2 plays a significant role in the occurrence and development of various diseases, such as cancer [7–10], myelodysplastic syndromes [11], diabetes [12, 13], and neurodegenerative disease [14, 15]. Therefore, the function and regulatory mechanism of NRF2 in cells have attracted wide attention and are considered key factors in maintaining cellular homeostasis and adapting to environmental changes. A thorough understanding of NRF2’s regulatory mechanisms and its involvement in disease pathogenesis is crucial for elucidating disease mechanisms and identifying potential therapeutic targets.

Post-translational modifications (PTMs) refer to the chemical modification process that proteins undergo after synthesis, which can regulate the activity, stability, and subcellular localization of proteins. In organisms, the diversity and complexity of PTMs of proteins provide the basis for the precise regulation of cellular functions [16]. Phosphorylation, ubiquitination, and acetylation are common forms of PTMs that interact and collectively regulate protein function and metabolism within the cell. These modifications play a critical role in key biological processes, including cell growth, differentiation, and stress response [17].

Epigenetic modifications refer to the regulation of gene expression through chemical modifications without altering the DNA sequence. These modifications influence gene transcription by altering chromatin structure and gene accessibility, thereby precisely regulating cellular functions and phenotypes in living organisms. The diversity and complexity of epigenetic modifications in organisms provide an essential foundation for the regulation of gene expression and cellular functions. Common forms of epigenetic modifications include DNA methylation, chromatin remodeling, and non-coding RNA regulation. These forms of epigenetic modifications interact with each other to jointly regulate gene expression, playing critical roles in processes such as cell differentiation, development, disease progression, and stress responses [17, 18].

**Fig. 1:**
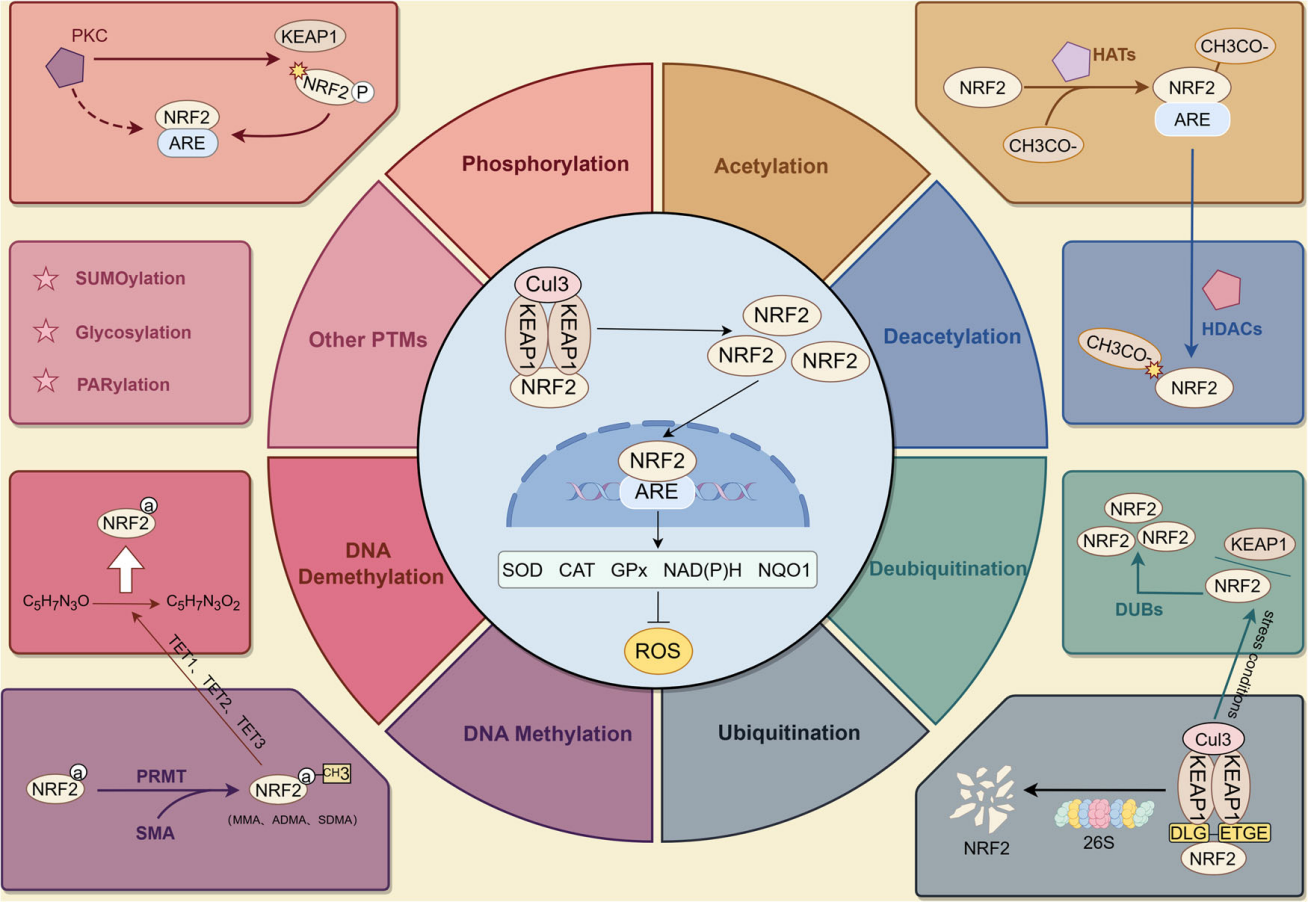
Post-translational modifications (PTMs) and epigenetic regulation of NRF2 in the antioxidant response. Under basal conditions, NRF2 is ubiquitinated by the Keap1-Cul3 complex for degradation. During oxidative stress, it dissociates from Keap1, translocates to the nucleus, and activates ARE-driven antioxidant genes like *SOD*, *CAT*, and *NQO1* to reduce ROS. Key PTMs include phosphorylation (activation), acetylation (enhanced transcription), deacetylation (repression), ubiquitination (degradation), and deubiquitination (stabilization), along with SUMOylation, glycosylation, and PARylation. Epigenetically, DNA methylation represses NRF2, while demethylation enhances its transcription.

As a transcription factor, NRF2 activity and stability are regulated by various PTMs and epigenetic modifications, which directly influence NRF2’s transcriptional activity and function. For example, IκB kinase (IKK) promotes the ubiquitination of NRF2 by activating the phosphorylation of CYLD, which in turn inhibits CYLD’s deubiquitination activity, further exacerbating oxidative stress damage in the kidneys of obesity-related nephropathy (ORN) [19]. In addition, dietary flavones promote the dissociation of NRF2 from Kelch-like ECH-associated protein 1 (Keap1) through two mechanisms: PKC-mediated phosphorylation of NRF2 and P62-mediated autophagic degradation of Keap1 [20]. Epigenetic modifications, such as DNA methylation and chromatin remodeling, also play critical roles in regulating NRF2 expression and function. The regulatory mechanisms governing these PTMs and epigenetic modifications are highly complex and multifaceted, influenced not only by the cellular environment but also by genetic variations, adding another layer of complexity to our understanding. Therefore, in-depth research into the relationships among NRF2, PTMs, and epigenetic modifications, as well as their roles in regulating NRF2 function, is crucial for elucidating the molecular mechanisms governing cellular redox homeostasis. Additionally, we have summarized the different PTMs and epigenetic modifications of NRF2 and their impacts on diseases (Fig. 1).

## Structure and function of NRF2

### Structural features of NRF2: basic structure and functional domains

**Fig. 2:**
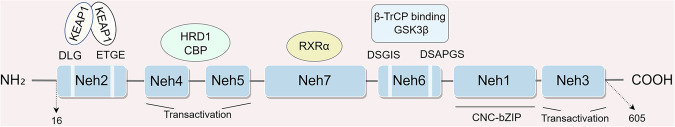
Domain structure of NRF2 and its regulatory interactions. The Neh2 domain binds Keap1 via the DLG and ETGE motifs, regulating NRF2 stability. Neh4 and Neh5 facilitate transcriptional activation by interacting with co-activators such as HRD1 and CBP. Neh7 inhibits NRF2 via interaction with RXRα, Neh6 promotes degradation through the DSGIS and DSAPGS motifs, interacting with β-TrCP and GSK3β. The Neh1 domain contains the CNC-bZIP motif for DNA binding and dimerization, and Neh3 supports transcriptional activation.

NRF2 is encoded by the nuclear factor erythroid 2-like 2 gene (NFE2L2) and is a member of the Cap’n’Collar (CNC) subfamily of basic leucine zipper (bZIP) transcription factors. This subfamily also includes nuclear factor erythroid-derived 2 (NFE2), NRF1, and NRF3, as well as two BTB and CNC homologous proteins (BTB and CNC homolog 1 (BACH 1) and BACH 2) [21]. Seven conserved regions of NRF2, known as the NRF2-ECH homology (Neh) structural domains, consist of 605 amino acids. These domains play distinct roles in regulating the transcriptional activity of NRF2 [22]. The Neh1 domain is located at the C-terminus of the NRF2 protein and contains the CNC and bZIP regions, which can bind to specific sequences on DNA to regulate the transcription of downstream genes. Following is the Neh2 region, also known as DLGex or ETGE region, which plays a crucial negative regulatory role in the control of NRF2. Under normal circumstances, NRF2 forms a complex with its inhibitory protein, Keap1 which regulates the stability and nuclear translocation of NRF2, thereby controlling its activity. The Neh2 region, serving as a critical binding domain with Keap1, subsequently governs the degradation and inhibition of NRF2 [23–25]. Currently, research on the Neh3 domain and the C-terminal transcriptional activation domains (Neh4 and Neh5) is limited. It is known that they interact with transcriptional repressor factors, exerting a negative regulatory effect on transcriptional activation of NRF2. While delving deeper into the functions and interactions of these domains, future research may uncover more detailed information about Neh3, Neh4, and Neh5 [26]. The Neh6 domain of NRF2 contains several degrons that are recognized by the E3 ubiquitin ligase β-transducin repeat-containing protein (β-TrCP). During oxidative stress, the inability of Keap1 to bind NRF2 leads to NRF2 accumulation, which subsequently activates the expression of antioxidant genes. Once the stress is relieved, Neh6-mediated degradation rapidly reduces NRF2 levels, restoring cellular homeostasis [27]. The Neh7 domain interacts with retinoic X receptor alpha (RXRα), thereby inhibiting NRF2 activity [28] (Fig. 2).

In summary, the structural characteristics of NRF2 highlight its multifunctional and intricated regulatory role in cellular stress responses. Through the interactions of these functional domains, NRF2 can sense and respond to oxidative stress signals both inside and outside the cell, thereby regulating the expression of a cascade of antioxidant and detoxification genes. This enables NRF2 to maintain cellular redox balance and stability in the survival environment.

### NRF2 regulating the antioxidant stress response

NRF2 is a crucial nuclear transcription factor that plays a key role in cellular antioxidant defense and protection. The functional mechanisms of NRF2 involve multiple levels, comprehensively regulating the cellular response to oxidative stress.

#### Combined with Keap1 for negative regulation

Under non-stress conditions, the protein levels of NRF2 are generally maintained at lower levels, which is attributed to the formation of a complex between NRF2 and its negative regulatory protein Keap1 in the cytoplasm. The interaction between the proteins relies on the binding between the C-terminal Kelch domain of Keap1 and the DLG and ETGE motifs within the Neh2 domain of NRF2. This interaction subsequently promotes partial ubiquitination of NRF2, which enhances its recognition by the ubiquitin-proteasome system. Ubiquitinated NRF2 is then degraded by the 26S proteasome in both the cytoplasm and nucleus, resulting in the negative regulation of NRF2 [29]. The ongoing degradation of NRF2 ensures that only the basal expression levels of NRF2 target genes are maintained, supporting essential housekeeping functions.

#### Oxidative stress induces the dissociation of the NRF2-Keap1 complex

When cells undergo oxidative stress, ionizing radiation, or chemical stimulation, it may lead to the generation of reactive oxygen species (ROS) or reactive oxygen free radicals within the cells, triggering an oxidative stress response. In this context, the structure of the NRF2-Keap1 complex may undergo alterations. Such structural changes could result in a conformational modification of the binding site between Keap1 and NRF2. Electrophiles and ROS may react with Keap1’s sensor cysteines, including cysteine 151 (C151), C273, and C288. This reaction weakens the binding between Keap1 and NRF2, allowing NRF2 to escape the negative regulation of Keap1 and avoiding ubiquitination and degradation. Therefore, the newly synthesized NRF2 accumulates in the cell, enabling it to exert its antioxidant and cellular protective functions [26].

#### Nuclear translocation and transcription of antioxidant genes

Upon dissociation, NRF2 translocates into the nucleus, where it binds to antioxidant response elements (AREs) located in the promoter regions of its target genes, thereby initiating their transcription. This process involves the nuclear localization signal, allowing NRF2 to traverse the nuclear pore and enter the cell nucleus. Upon binding to AREs in the nucleus, NRF2 interacts with other co-activators, such as the CBP/p300 protein, forming a complex that further activates the transcription of antioxidant and detoxification genes [30]. This orchestrated process regulates the expression of a series of antioxidant enzymes and detoxifying enzymes, including superoxide dismutase, catalase, glutathione peroxidase, NQO1, and others [31–33]. They play antioxidant and detoxification roles within the cell. This complex regulatory network ensures timely and effective responses to oxidative stress both inside and outside the cell, maintaining cellular redox balance. This contributes to maintaining the integrity of cell membranes, proteins, and nucleic acids, preserving the overall health of the cell.

Overall, NRF2 plays a crucial cellular protective role through the sensing of oxidative stress, nuclear translocation, binding to AREs, and the regulation of the expression of antioxidant and detoxification enzymes. This regulatory mechanism is irreplaceable in preventing cellular damage caused by oxidative stress and maintaining cellular homeostasis. A profound understanding of NRF2’s functional mechanisms contributes to the development of novel therapeutic strategies, particularly in addressing diseases associated with oxidative stress.

## PTMs of NRF2

PTMs, such as phosphorylation, acetylation, ubiquitination, methylation, and glycosylation, among others, are largely reversible processes. These modifications regulate various aspects of target proteins, including their activity, stability, interactions with other proteins, and intracellular localization [34]. Protein PTMs play a crucial role in allowing cells and organisms to respond quickly and flexibly to various stresses. In recent years, the impact of PTMs on NRF2 has become increasingly prominent [35].

### Phosphorylation

Phosphorylation is a widely distributed regulatory mechanism present in living organisms. This modification is orchestrated by a group of enzymes, predominantly kinases, which catalyze the addition of phosphate groups to specific protein residues. The phosphorylation process typically occurs on serine, threonine, or tyrosine residues of proteins, where hydrogen atoms on these residues are replaced by phosphate groups [36].

Protein Kinase C (PKC) is a crucial family of protein kinases and a part of the Mitogen-Activated Protein Kinase (MAPK) family. PKC regulates various biological processes through phosphorylation, including cell proliferation, differentiation, apoptosis, cell motility, and signal transduction. The PKC family is composed of various serine/threonine kinase subtypes. These include conventional PKC isoforms (PKCα, PKCβI, PKCβII, and PKCγ), novel PKC isoforms (PKCδ, PKCε, PKCη, and PKCθ), and atypical PKC isoforms (PKCζ and PKCι/λ) [37]. All PKC isoforms share common structural characteristics, which include an N-terminal regulatory domain, a hinge region, a conserved kinase domain, and a C-terminal tail domain [38]. The activity of PKC is regulated by various factors, including signaling molecules on the cell membrane, secondary messengers, and interactions with other proteins. For instance, Diacylglycerol can directly bind to PKC, inducing a conformational change in PKC that results in the generation of phosphorylated PKC, subsequently activating the enzyme [39].

In the study of NRF2, PKC serves as a crucial regulatory factor involved in the phosphorylation process of NRF2 [40]. After activation, PKC selectively phosphorylates NRF2, leading to its dissociation from Keap1 and relieving Keap1-mediated negative regulation of NRF2. Upon translocation into the nucleus, NRF2 forms a complex with ARE, enhancing cellular resistance to oxidative stress. This plays a crucial role in regulating the basal expression and antioxidant induction of NQO1 and other detoxification genes [41].

Phosphorylation of NRF2 by PKC has been shown to play a critical role in various biological processes. Phorbol 12-myristate 13-acetate (PMA) is a well-established activator of PKC. It can enhance the activity of NRF2 in HepG2 human liver cancer cells. This effect can be abolished by the PKC-specific inhibitor staurosporine. Moreover, treatment with tert-butylhydroquinone (tBHQ) or PMA has been shown to promote PKC-mediated phosphorylation of NRF2 in HepG2 cells, leading to enhanced nuclear localization of NRF2. These findings indicate that NRF2 is a potential target of PKC regulation [42]. Further studies have shown that PKC directly phosphorylates Ser 40 within the Neh2 domain of NRF2, which is necessary for the dissociation of NRF2 from Keap1 [41]. Further research can attempt to identify and validate these sites, exploring their role in regulating NRF2 activity and function.

In a rat model of ischemia-reperfusion injury, the cardioprotective effects induced by postconditioning were found to be associated with PKC activation and NRF2 phosphorylation. Additionally, inhibition of PKC was shown to reduce NRF2 phosphorylation and transcriptional activity, along with the expression and activity of NRF2-regulated antioxidant proteins [43]. Furthermore, a study in 2021 demonstrated that propofol can reduce myocardial ischemia/reperfusion (I/R) injury by activating the PKC/ NRF2 pathway in H9C2 cells and the rat Langendorff model [44]. Chemotherapeutic agents, such as doxorubicin (DOX), can result in cardiomyopathy and potentially life-threatening arrhythmias in cancer patients. The Pt2/EP1 axis has been shown to inhibit ferroptosis in DOX-induced cardiomyocytes through the PKC/NRF2 signaling pathway, thereby protecting the heart from DIC [45]. P450 2A6 (CYP2A6) metabolizes nicotine, leading to oxidative stress and the generation of harmful metabolites, which can cause liver damage and lung cancer. Ethanol (EtOH) modulates CYP2A6 expression by activating the PKC/MEK/NRF2 pathway, potentially increasing HIV-1 replication in smokers. This study holds clinical significance for HIV-positive individuals who abuse alcohol and tobacco concurrently [46]. Recently, a study by Maha Abdelmonem *et al*. found that modulating the PKC/NRF2/Bcl-2 signaling pathway can enhance the clinical efficacy of sitagliptin, improving diabetes-induced testicular dysfunction [47]. The β-amyloid (Aβ) peptide contributes to neurotoxicity by inducing oxidative stress and inflammatory responses, thereby promoting the progression of Alzheimer’s disease (AD). Maackiain, extracted from peony root, exhibits antioxidative, anti-osteoporotic, anti-tumor, and immunomodulatory properties. Maackiain promotes NRF2 activation through the PKC signaling pathway, preventing Aβ-induced oxidative stress and cellular damage in PC12 cells, indicating its potential for treating AD [48]. In kidney diseases, gentamicin can induce nephrotoxicity, leading to impaired renal function, while Riceberry bran extract (RBBE) can initiate protective mechanisms in the kidneys via regulation of the PKC/NRF2 antioxidant defense pathway [49]. Overall, phosphorylation of NRF2, as a dynamic and reversible protein modification, is important for the regulation of signaling and biological processes inside and outside the cell (Table 1).

**Table 1:** Targeted drugs for NRF2 in phosphorylation.

Drug	Function	References
PMA	NRF2 upregulation	[42]
tBHQ	NRF2 upregulation	[42]
Propofol	Activate the PKC/NRF2 pathway	[44]
DOX	Activate the PKC/NRF2 pathway	[45]
EtOH	Activate the PKC/MEK/NRF2 pathway	[46]
Sitagliptin	modulating the PKC/NRF2/Bcl-2 signaling pathway	[47]
Maackiain	Activate the PKC/NRF2 pathway	[48]
RBBE	Activate the PKC/NRF2 pathway	[49]

### Acetylation

Acetylation, a pivotal post-translational modification, involves the covalent addition of acetyl (CH3CO-) to specific amino acid residues, predominantly lysine ε-amino groups, resulting in a variety of functions. This modification can neutralize the positive charge of lysine, affecting protein-protein interactions and stability [50]. Notably, lysine acetylation plays a crucial role in regulating gene expression by modulating the chromatin structure, as acetylated histones are often associated with active transcription [51]. Additionally, it serves as a signaling mechanism, influencing cellular pathways, such as cell cycle control, histone ubiquitination, and DNA repair [52]. The process of acetylation is catalyzed by acetyltransferases and can be reversed by deacetylases, thereby maintaining a critical balance essential for cellular homeostasis and the regulation of normal biological functions [53].

NRF2 acetylation represents a key post-translational modification, characterized by the addition of acetyl groups (CH3CO-) to specific lysine residues on the NRF2 protein within the cell. This modification is generally mediated by enzymes known as histone acetyltransferases (HATs). Conversely, histone deacetylases (HDACs) are capable of removing acetyls from NRF2, thereby reducing its acetylation level and attenuating its transcriptional activity and stability. Both HATs and HDACs collectively regulate the acetylation level of NRF2. Acetylation occurs in multiple functional domains of NRF2, particularly within the transactivation domain and other critical structural regions [54].

Acetylation strengthens the interaction between NRF2 and its transcriptional co-activators, facilitating the transcriptional activation of genes involved in antioxidant and detoxification processes. This enhancement is attributed to acetylation-induced alterations in the binding properties of NRF2 with transcription factors, increasing the affinity of NRF2 for DNA binding, and consequently driving the expression of relevant genes. This modification also enhances the stability of the NRF2 protein, prolonging its presence within the cell [55]. By reducing the rate of NRF2 ubiquitination and degradation, it amplifies its functions. This implies that NRF2 can engage in antioxidant and detoxification reactions for an extended period, providing sustained cellular protection. Furthermore, under acetylation conditions, the nuclear localization of NRF2 is increased. This facilitates its interaction with basic region leucine zipper proteins at AREs thereby enhancing the transcription of antioxidant genes. Conversely, under deacetylation conditions, NRF2 localization shifts towards the cytoplasm rather than the nucleus, resulting in its dissociation from AREs and termination of transcription [56]. N-α-acetyltransferase 10, also known as Arrest defective 1 (ARD1), is an N-terminal acetyltransferase. In colorectal cancer, it has been found that the overexpression of ARD1 can enhance the acetylation level of NRF2, thereby affecting NRF2’s transcriptional activity and nuclear localization. As a result, targeting the ARD1-NRF2 axis could offer a potential therapeutic strategy for colorectal cancer [57]. The histone acetyltransferase MOF, a member of the MYST family, is overexpressed in human non-small cell lung cancer (NSCLC) tissues, influencing both disease progression and prognosis. MOF-mediated acetylation enhances NRF2 nuclear retention and downstream gene transcription, playing a critical role in antioxidant responses and drug resistance, and regulating tumor growth and resistance through an NRF2-dependent mechanism [58].

### Deacetylation

The intracellular processes of acetylation and deacetylation are usually in dynamic equilibrium, with dual regulatory mechanisms for protein function and regulation. Contrary to acetylation, deacetylation is catalyzed by deacetylases, which act to remove acetyls from lysine residues of proteins. This modification process regulates the structure and function of proteins, usually by making them more compact or affecting their stability and activity within the cell [59]. The effects of acetylation reactions on NRF2 are multifaceted and collectively regulate the function of NRF2 in cellular responses to oxidative stress and disease progression, affecting its potential in cytoprotective and therapeutic targets.

HDACs are a class of enzymes that regulate gene expression by removing acetyls from histones and play an important role in cell regulation and disease development [60]. Upregulation of Histone deacetylase 3 (HDAC3) in pulmonary fibrosis can lead to NRF2 inhibition, and by selectively inhibiting HDAC3 (e.g., using the drug RGFP966), the inhibitory effect on NRF2 can be reduced. This intervention can effectively restore NRF2-mediated antioxidant responses in disease models and help attenuate pathological processes in related diseases such as pulmonary fibrosis [61]. HDAC3 has also been demonstrated to improve endothelial dysfunction in type 2 diabetes mellitus (T2DM)-associated cardiovascular disease by mitigating inflammation and oxidative stress. This occurs through the regulation of NRF2, a nuclear factor responsible for modulating the expression of genes related to anti-inflammatory responses and cell survival in the vascular endothelium [62]. Furthermore, enhanced permeability of the blood-brain barrier is a critical neurovascular complication of T2DM, which disrupts CNS homeostasis and impairs its function. Inhibition of HDAC3 can indirectly promote NRF2 acetylation and nuclear translocation, thereby improving T2DM-induced blood-brain barrier permeability [63]. Nuclear Factor kappa-light-chain-enhancer of activated B cells (NF-κB) constitutes a family of transcription factors that play pivotal regulatory roles within the cell. In an I/R injury model, NF-κB was found to inhibit NRF2-ARE antioxidant signaling via HDAC3 and promote oxidative stress-induced cell death [64]. HDAC5 inhibits NRF2-dependent antioxidant gene expression and plays an important role in regulating redox homeostasis in cardiomyocytes [65]. Furthermore, studies on HDAC6 have shown that inhibiting HDAC6 can protect mice from experimental stroke-induced brain injury by regulating oxidative stress through activation of the NRF2/HO-1 pathway [66]. HDACi are compounds that can inhibit the activity of HDAC. By inhibiting HDAC, HDACi can increase the acetylation levels of proteins. Thereby altering chromatin structure and activating gene expression. HDACi shows neuroprotective effects in animal models of retinal I/R injury. These mechanisms of action are primarily achieved by promoting the acetylation and nuclear translocation of NRF2, which in turn enhances the function of NRF2 in antioxidant and anti-inflammatory responses [67].

Astilbin is a flavonoid compound derived from plants, while SIRTuin 1 (SIRT1) is a member of the SIRTuin family of NAD-dependent lysine deacetylases. Astilbin interacts with SIRT1 to promote the deacetylation of NRF2, thereby inhibiting oxidative stress in grass carp hepatocytes. This mechanism helps alleviate mitochondrial apoptosis induced by 3,3′,4,4′, and 5-pentachlorobiphenyl, which is triggered by imbalances in mitochondrial dynamics and energy metabolism [68]. The novel SIRT1 activator SRT2104 may offer protection against diabetic nephropathy by modulating the SIRT1/P53/NRF2 axis, thereby enhancing renal antioxidant activity in diabetic conditions [69]. Ellagic acid (EA), a natural antioxidant, mitigates aging-related oxidative damage in the kidney by upregulating the mRNA and protein expression of SIRT1 and NRF2, while also promoting the deacetylation of NRF2 protein [70]. Inflammation and oxidative stress are recognized as critical factors in the pathogenesis of depression. Edaravone, a free radical scavenger, exhibits strong antidepressant and anxiolytic effects through the activation of the SIRT1/NRF2/HO-1/Gpx4 axis [71]. In renal ischemia/reperfusion (I/R) injury, fucoidan activates the SIRT1/NRF2/HO-1 signaling pathway to reduce oxidative stress-induced apoptosis [72]. Astaxanthin mitigates oxidative stress and neuronal death via the SIRT1/NRF2/Prx2/ASK1/p38 signaling pathway, enhances neurological function, and provides neuroprotection following traumatic brain injury [73]. In studies of myocardial ischemia/reperfusion injury, the cardioprotective effect of SIRT1 was found to be associated with deacetylation of NRF2 [74]. Overexpression of MicroRNA-126 (miR-126) mitigates oxygen-glucose deprivation/reperfusion injury by reducing oxidative stress and inflammation through the activation of the SIRT1/NRF2 signaling pathway in human umbilical vein endothelial cells [75]. In aged mice, the adaptive homeostasis regulated by NRF2 is diminished in the liver. Nicotinamide Mononucleotide(NMN) supplementation restores redox balance in the liver through the SIRT3-NRF2 axis, protecting the aged liver from oxidative stress-induced damage [76]. SIRT5 regulates the NRF2/HO-1 pathway in a ROS-dependent manner to inhibit cisplatin (CDDP)-induced DNA damage, thereby promoting CDDP resistance in ovarian cancer (OC) [77]. SIRT6 may also mitigate vascular inflammation through its deacetylase activity and NRF2-dependent signaling pathway [78]. Ginsenoside Rc, the main active ingredient isolated from ginseng, increases the deacetylase activity of SIRT6, thereby decreasing the level of acetylated NRF2 and increasing the stability of NRF2, which in turn attenuates hepatocellular injury and oxidative stress in alcoholic liver disease [79]. Sodium butyrate (NaB), a histone deacetylase inhibitor, increases NRF2 expression and alleviates oxidative stress and insulin resistance induced by a high-fat diet [80]. In a study of chronic kidney disease (CKD), overexpression of SIRT6 was found to activate the NRF2/HO-1 pathway. This attenuates angiotensin II (Ang II)-induced oxidative stress, DNA double-strand breaks, and podocyte damage, which is closely related to the pathogenesis of CKD [81] (Table 2).

**Table 2:** Targeted drugs for NRF2 in Acetylation.

Drug	Function	References
RGFP966	NRF2 upregulation	[61]
HDACi	NRF2 upregulation	[67]
Astilbin	NRF2 upregulation	[68]
SRT2104	Enhancing the SIRT1/P53/NRF2 pathway	[69]
EA	NRF2 upregulation	[70]
Edaravone	Activate the SIRT1/NRF2/HO-1/Gpx4 pathway	[71]
Fucoidan	Activate the SIRT1/NRF2/HO-1 pathway	[72]
Astaxanthin	Enhancing the SIRT1/NRF2/Prx2/ASK1/p38 pathway	[73]
NMN	Enhancing the SIRT3-NRF2 pathway	[76]
Ginsenoside Rc	NRF2 upregulation	[79]
NaB	NRF2 upregulation	[80]

### Ubiquitination

Ubiquitination is an intracellular protein modification process that regulates various biological processes by covalently attaching a 76-amino-acid polypeptide called ubiquitin to target proteins. [82]. Initially, ubiquitin-activating enzyme E1 binds with ATP to activate the ubiquitin molecule, forming a thioester bond between the carboxyl group of ubiquitin’s C-terminal and the cysteine residue of the E1 enzyme. Then, the activated ubiquitin is transferred to the cysteine residue of ubiquitin-conjugating enzyme E2, with the formation of a thioester bond as well, and preparing for the subsequent transfer. Finally, ubiquitin ligase E3 identifies specific target proteins and transfers ubiquitin from E2 to the lysine residue on the target protein. E3 enzyme ensures the specificity of the ubiquitination process by recognizing particular proteins [83]. Such modifications can form mono-ubiquitination, poly-ubiquitination, and poly-ubiquitin chains. Poly-ubiquitin chains are particularly important, as they often label target proteins to be sent to the proteasome for degradation, which is a major mechanism by which cells remove unwanted or damaged proteins. Additionally, ubiquitination plays various crucial roles within the cell, extending beyond protein degradation to encompass signal transduction [84], DNA repair [85], cell cycle regulation [78, 79], and the modulation of protein localization and activity [86], among other biological processes. These functions make ubiquitination one of the key regulatory mechanisms within the cell, essential for maintaining normal cellular function and vital activities.

Keap1 is a cytoplasmic protein with a Kelch repeat domain for binding NRF2 and a BTB (Bric-a-brac, Tramtrack, Broad complex) domain for binding Cullin3 (Cul3). As an inhibitor of NRF2, Keap1 recognizes and binds to the ETGE and DLG motifs of NRF2 via its Kelch repeat domain, thereby continuously suppressing and promoting the degradation of NRF2 [87]. Cul3 is the scaffold protein of the E3 ligase complex and can form a complex with RBX1 and Keap1. RBX1 is responsible for ubiquitin transfer. Keap1 binds to Cul3 via its BTB domain, forming the Keap1-Cul3-RBX1 E3 ligase complex, which plays a crucial role in the ubiquitination of NRF2 [88, 89]. Under normal conditions, NRF2 is recognized and ubiquitinated by the Keap1-Cul3 E3 ligase complex, marking it for proteasomal degradation and maintaining its low levels in the cell. This mechanism ensures that in the absence of oxidative stress or other stimuli, the expression of NRF2-dependent antioxidant genes is suppressed [88, 90]. Under stress conditions, such as oxidative stress, the levels of ROS in the cell increase, leading to modifications of certain cysteine residues in Keap1. These modifications alter its conformation, preventing Keap1 from effectively binding to the ETGE and DLG motifs of NRF2. Consequently, NRF2 dissociates from Keap1, escapes ubiquitination and degradation, accumulates in the cytoplasm, and ultimately translocates to the nucleus [85, 91, 92]. In summary, the Keap1-Cul3 E3 ligase complex regulates the levels and activity of NRF2 through ubiquitination and degradation mechanisms. This ensures that NRF2 remains at low levels under normal conditions but can be rapidly activated under stress conditions, helping the cell cope with oxidative damage and other harmful stimuli.

**Fig. 3:**
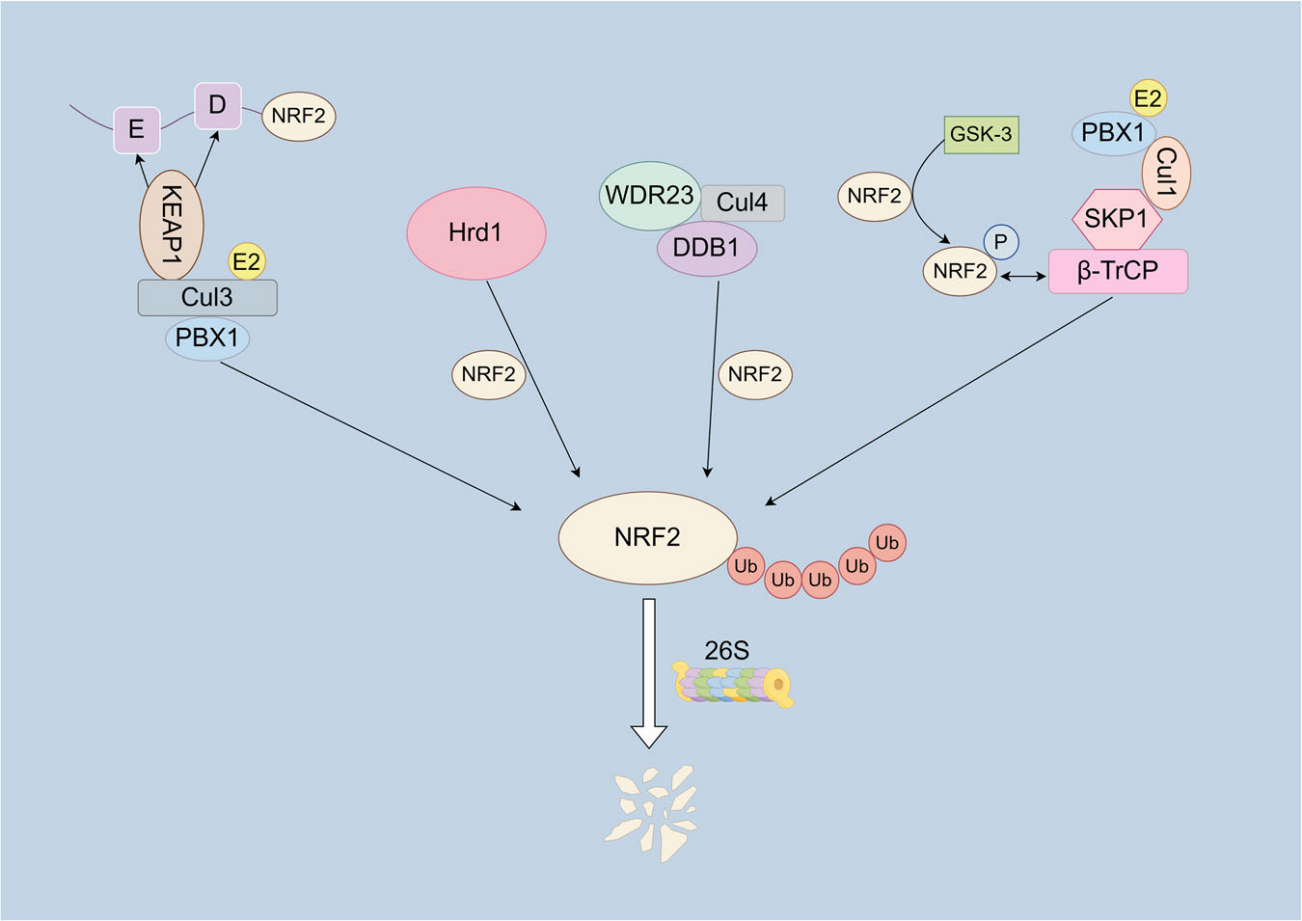
Ubiquitination pathways regulating NRF2 degradation. The KEAP1-Cul3 complex ubiquitinates NRF2 via the Cul3-PBX1 complex, targeting it for proteasomal degradation. The β-TrCP-Cul1 complex, activated by GSK-3-mediated phosphorylation of NRF2, promotes its ubiquitination and degradation. The Hrd1 pathway directly ubiquitinates NRF2 through the Hrd1 E3 ligase. The WDR23-Cul4-DDB1 complex facilitates NRF2 ubiquitination and degradation.

In addition to Keap1-mediated ubiquitination and degradation, NRF2 stability is regulated by other mechanisms. β-TrCP, an F-box protein, facilitates NRF2 degradation via the Skp1-Cullin-F-box E3 ligase complex. β-TrCP specifically recognizes phosphorylated sites on NRF2, targeting it for ubiquitination and subsequent proteasomal degradation [92, 93]. Similarly, glycogen synthase kinase-3 (GSK-3) can phosphorylate specific sites on NRF2, promoting its recognition and binding by β-TrCP, thereby mediating the ubiquitination and degradation of NRF2. Additionally, HMG-CoA reductase degradation protein 1 (Hrd1) is an E3 ligase primarily involved in the endoplasmic reticulum-associated degradation (ERAD) pathway. Hrd1 can recognize and bind NRF2, directing it to the ERAD pathway for ubiquitination and proteasomal degradation [55]. Finally, WD repeat domain 23 (WDR23) can form a complex with Damage-Specific DNA Binding Protein 1 (DDB1) and Cullin 4, recognizing and promoting the ubiquitination of NRF2, thereby regulating its stability [21] (Fig. 3).

It is well known that the activation of NRF2 can enhance the antioxidant defenses of cancer cells, helping them resist oxidative stress and increasing their survival and proliferation. This enhanced antioxidant capacity also enables cancer cells to develop resistance to chemotherapeutic drugs [94]. On the other hand, the ubiquitination of NRF2 exerts a certain degree of anti-cancer effect. Its ubiquitination and degradation can limit the activation of antioxidant genes, thereby reducing the survival and proliferation of cancer cells. By elevating oxidative stress, NRF2 degradation induces apoptosis in cancer cells and increases their sensitivity to chemotherapeutic agents, thereby enhancing the effectiveness of chemotherapy [95]. 3-Hydroxybutyrate dehydrogenase 2 (BDH2) is a member of the short-chain dehydrogenase/reductase family and has been identified as a significant tumor suppressor in gastric cancer (GC). It promotes NRF2 ubiquitination through the Keap1/NRF2/ARE signaling pathway, induces ROS accumulation, and inhibits the phosphorylation of AktSer473 and mTORSer2448. By mediating the PI3K/Akt/mTOR pathway, it inhibits the growth of GC [96]. Glutathione-S-transferases are phase II detoxification enzymes that maintain redox homeostasis. In GC tissues, the expression of Glutathione-S-Transferase (GST) family members is significantly downregulated, particularly Glutathione-S-transferase mu 3 (GSTM3). The significant downregulation of GSTM3 is associated with independent prognosis in GC and inhibits GC cell proliferation and migration. It has also been found that the expression of GSTM3 and NRF2 is often positively correlated. GSTM3 is transcriptionally regulated by the NRF2/Keap1 signaling pathway and interacts with Cullin-associated and Neddylation-dissociated 1 (CAND1). This interaction impairs the association between NRF2 and Keap1, inhibiting NRF2 ubiquitination and subsequent degradation, thereby promoting NRF2 activation [97]. The combination therapy of metformin and CDDP weakens ERK-mediated phosphorylation of NRF2, enhances its polyubiquitination, and promotes its proteasomal degradation, thereby significantly reducing the levels of NRF2 in A549/DDP and H838 NSCLC cells, promoting ROS-mediated apoptosis. Fluctuations in NRF2 levels predict the chemotherapy response and survival outcomes of NSCLC patients receiving neoadjuvant chemotherapy, and unaltered NRF2 expression correlates with poor survival rates and chemotherapy resistance in NSCLC patients [98]. In the study by Di Zhang et al., a novel small molecule, MSU38225, was discovered to inhibit the NRF2 pathway. Treatment with MSU38225 enhanced the ubiquitination of NRF2, reducing its protein levels. It also downregulated NRF2 transcriptional activity, decreased the expression of downstream targets such as NQO1, GCLC, GCLM, AKR1C2, and UGT1A6, and inhibited the growth of human lung cancer cells. These findings suggest that MSU38225 could serve as an adjuvant therapy, enhancing the sensitivity of lung cancer patients to chemotherapy [99]. Progestin and AdipoQ Receptor 4 (PAQR4), a member of the PAQR family localized in the Golgi apparatus, competitively binds to NRF2 when highly expressed, thereby enhancing the stability and nuclear translocation of NRF2. This interaction inhibits Keap1-mediated ubiquitination and degradation of NRF2, resulting in the upregulation of antioxidant genes and contributing to chemotherapy resistance in NSCLC [100]. In other cancer processes, the ubiquitination of NRF2 has also been found to have potential impacts. The ubiquitination of NRF2 may also influence drug resistance in OC [101]. Danshen-derived compound 1 (DHT) is a bioactive compound found in Danshen with cytotoxic effects against various malignant tumors. In ovarian tumor (OT) tissues, DHT enhances the binding of NRF2 to Keap1, resulting in potentiated ubiquitination and degradation of NRF2, thereby activating oxidative stress and exhibiting anti-tumor effects [102]. In OC treatment, the natural compound β-sitosterol (SIT) has demonstrated the potential to strengthen the interaction between NRF2 and Keap1, promoting NRF2’s ubiquitin-dependent degradation and subsequently diminishing the transcriptional activation of downstream antioxidant genes. By disrupting the antioxidant defense mechanisms, SIT induces a marked accumulation of ROS in OC cells. This upregulates Phosphatase and Tensin Homolog (PTEN) and inhibits the phosphorylation of AKT. Suppression of AKT signaling affects key pathways involved in the cell cycle, survival, apoptosis, migration, and invasion, ultimately resulting in OC cell death [103]. Brucein D, a naturally occurring quassinoid, enhances the sensitivity of pancreatic ductal adenocarcinoma (PDAC) cells to gemcitabine (GEM) by promoting the ubiquitin-proteasome-dependent degradation of NRF2 and suppression of the NRF2 signaling pathway [104]. Teng Xu and colleagues identified that, in head and neck squamous cell carcinoma (HNSCC), the DLG motif within tumor necrosis factor-α-induced protein 2 (TNFAIP2) competes with the Kelch domain of Keap1, thereby inhibiting the ubiquitin-proteasome-mediated degradation of NRF2. This results in NRF2 accumulation and CDDP resistance [105]. Research on osteosarcoma treatment has revealed that DDRGK domain-containing protein 1 (DDRGK1) inhibits the ubiquitin-proteasome-mediated degradation of NRF2 by competitively binding to Keap1. This interaction reduces NRF2 stability, resulting in the accumulation of ROS, which promotes apoptosis in cancer cells and enhances their sensitivity to DOX and etoposide [106]. Baicalin has been found to inhibit the growth of oxidative stress cells by inducing the ubiquitin-mediated degradation of NRF2, thereby affecting its stability. This inhibition leads to the downregulation of NRF2 downstream targets Glutathione Peroxidase 4 (GPX4) and SLC7A11, resulting in the induction of ferroptosis in tumor cells [107]. Additionally, in oxidative stress cells, Tripartite Motif Containing 22 (TRIM22) has been found to interact with Keap1 and accelerate the ubiquitination and degradation of NRF2, thereby inhibiting oxidative stress progression and activating ROS/AMPK/mTOR/autophagy signaling pathways. Therefore, targeting the TRIM22/NRF2 axis may represent a promising therapeutic strategy for the treatment of oxidative stress [108]. Notably, Brusatol, a natural NRF2 inhibitor, enhances the binding of KEAP1 to NRF2, promotes NRF2 ubiquitination, and accelerates its proteasomal degradation, thereby suppressing NRF2-driven antioxidant function and sensitizing cancer cells to oxidative stress. For example, Brusatol specifically reduces NRF2 protein expression through ubiquitin-mediated degradation of NRF2, thereby enhancing the sensitivity of AML cells to cytarabine [109]. In the study of endometrial cancer (EC), we found that Brusatol sensitizes the cancer to progestins by inhibiting the NRF2-TET1-AKR1C1 pathway, which reduces AKR1C1 expression and decreases progesterone metabolism [110]. Additionally, Brusatol significantly inhibits the proliferation of HER2-positive cancer cells by suppressing the NRF2/HO-1 and HER2-AKT/ERK1/2 signaling pathways. Its combination with trastuzumab further enhances anti-tumor activity, inducing ROS accumulation and promoting apoptosis in SK-OV3 and BT-474 cells, thereby significantly improving the therapeutic effect [111] (Table 3).

**Table 3:** Drugs targeting the KEAP1-NRF2 pathway.

Drug or target	Cancer	Mechanism	Impact	Reference
BDH2	GC	Promotes ubiquitination of NRF2	Exerts tumor-suppressive effects	[96]
GSTM3	GC	Inhibition of NRF2 ubiquitination and degradation.	Inhibit the proliferation and migration of GC cells	[97]
PAQR4	NSCLC	Inhibition of NRF2 ubiquitination and degradation.	Enhancing the expression of antioxidant genes correlates with chemotherapy resistance.	[100]
DHT	OT	Promotes ubiquitination of NRF2	Activating oxidative stress, Exhibiting anti-tumor effects	[102]
SIT	OC	Trigger NRF2 ubiquitin-dependent degradation.	Promoting OC cell death	[103]
Brucein D	PDAC	Promote ubiquitin-proteasome-dependent degradation of NRF2.	Enhancing the sensitivity of PDAC cells to GEM	[104]
TNFAIP2	HNSCC	Inhibit ubiquitin-proteasome-mediated degradation of NRF2.	Developing resistance to CDDP	[105]
DDRGK1	OS	Inhibit ubiquitin-proteasome-mediated degradation of NRF2.	Enhancing osteosarcoma sensitivity to DOX and etoposide.	[106]
TRIM22	OS	Promote ubiquitination and degradation of NRF2.	Inhibiting the progression of osteosarcoma.	[108]
Brusatol	AML	Promotes ubiquitination of NRF2	Enhancing the sensitivity of AML cells to cytarabine.	[109]
Brusatol	EC	Promotes ubiquitination of NRF2	Enhancing the sensitivity of endometrial cancer to progestins.	[110]
Brusatol	HER2-positive cancer	Promotes ubiquitination of NRF2	Inhibiting the proliferation of HER2-positive cancer cells.	[111]

Similarly, in the development of other diseases, the ubiquitination of NRF2 also plays a potential role. Lumbar spinal stenosis (LSS) can result in irreversible nerve damage and dysfunction, primarily characterized by hypertrophy and fibrosis of the ligamentum flavum (LF). In LSS, the expression of the E3 ubiquitin ligase Smurf1 is markedly elevated, which promotes fibrosis and oxidative stress in LF cells, contributing to the progression of LSS by facilitating the ubiquitination and degradation of NRF2 [112]. Ischemia-reperfusion (I/R) injury is linked to endoplasmic reticulum stress (ERS) and mitochondrial dysfunction, resulting in oxidative stress-induced acute kidney injury. X-box binding protein 1 (XBP1), a key regulator of the ERS response, along with HRD1, shows elevated expression during I/R injury. This upregulation promotes the interaction between HRD1 and NRF2, inducing the ubiquitination and degradation of NRF2. The downregulation of NRF2 subsequently induces ROS production, impairing renal function. Therefore, downregulation of XBP1 can effectively protect the kidneys from I/R-induced injury [113]. Furthermore, 1,25-dihydroxyvitamin D [1,25-(OH)_2_D] can enhance NRF2 accumulation by inhibiting its ubiquitin-proteasome-mediated degradation. Consequently, preventing NRF2 degradation may help mitigate age-related osteoporosis resulting from 1,25-(OH)_2_D deficiency [114]. Nerve growth factor (NGF), a member of the neurotrophin family, regulates pain perception in various acute and chronic pain conditions. Sorting nexin 25 (SNX25) in dermal macrophages inhibits the ubiquitination and proteasomal degradation of NRF2, thereby regulating the production of NGF in dermal macrophages. The deletion of SNX25 accelerates the degradation of NRF2 and reduces NGF expression, leading to decreased pain sensitivity [115]. Metformin, a first-line antidiabetic medication, has been demonstrated to increase the radiosensitivity of NSCLC cells. It enhances NRF2 ubiquitination and proteasomal degradation through a mechanism that is independent of Keap1. This reduction in NRF2 levels results in decreased transcription of downstream antioxidant proteins, suppression of DNA damage repair pathway initiation, and disruption of G2/M phase arrest following radiation exposure [116]. Over the past decade, the number of patients with ORN has doubled. Research has shown that IKK phosphorylates CYLD to inhibit its deubiquitination activity, thereby increasing NRF2 ubiquitination [19]. Targeting NRF2 ubiquitination provides multiple therapeutic strategies to combat cancer and other diseases. First, inhibiting NRF2 activation, such as using Deubiquitinase 3 (DUB3) inhibitors, promotes NRF2 ubiquitination and degradation. This reduces the activation of antioxidant genes, limiting cancer cell survival and proliferation while enhancing their sensitivity to chemotherapeutic drugs. These approaches are mainly used for colorectal [117].

### Deubiquitination

Contrary to ubiquitination, deubiquitination refers to the process of removing ubiquitin molecules from proteins. This process is catalyzed by deubiquitinating enzymes (DUBs) and is a crucial regulatory mechanism in post-translational modification of proteins. DUBs are classified into five major categories: ubiquitin carboxyl-terminal hydrolases, ubiquitin-specific proteases, metalloproteases, OTU domain-containing DUBs, and JAMM domain-containing DUBs [118]. Deubiquitination can reverse the effects of ubiquitination, thereby regulating protein stability, preventing proteasomal degradation, and extending protein lifespan. It also modulates signal transduction by influencing key proteins in cellular signaling pathways, affecting cell proliferation, differentiation, and apoptosis. Additionally, deubiquitination impacts the localization and activity of proteins within the cell, altering interactions with other molecules and thus regulating protein function [119]. Deubiquitination, akin to ubiquitination, is essential for maintaining cellular homeostasis and regulating a wide array of physiological processes. This regulation occurs through a complex network of mechanisms that ensure proper protein function and turnover within the cell.

The deubiquitinase Ubiquitin Carboxyl-Terminal Hydrolase L3 (UCHL3) has been identified as an oncogenic factor in various cancers. In cervical cancer studies, it has been found that UCHL3 promotes the development and metastasis of cervical cancer by stabilizing NRF2 through deubiquitination [120]. Ubiquitin-specific protease 11 (USP11) stabilizes NRF2 protein levels through deubiquitination. Depletion of USP11 inhibits cell proliferation and induces ROS-mediated stress-induced ferroptosis, an effect that can be mitigated by overexpression of NRF2. Immunohistochemical analysis of lung tissue microarrays demonstrated elevated USP11 expression in NSCLC patients, which exhibited a positive correlation with NRF2 expression. USP11 promotes NRF2 stabilization by mediating its deubiquitination, thereby playing a pivotal role in regulating cell proliferation and ferroptosis [121]. Inhibition of the NRF2-GSH axis sensitizes the small molecule Ras-Selective Lethal 3 (RSL3) to induce ferroptosis in Kirsten Rat Sarcoma Viral Oncogene Homolog Lung Adenocarcinoma cells in vitro. This process occurs through the direct binding of RSL3 to USP11, resulting in its inactivation and subsequent induction of ubiquitination and degradation of NRF2 protein in these cells [122]. Jing Cui et al. found that in pancreatic cancer patients, the expression of ubiquitin-specific protease 8 (USP8) is increased, and USP8 stabilizes NRF2 expression by deubiquitinating K48-linked polyubiquitin chains on NRF2. The chemotherapy drug GEM further induces USP8 expression, leading to increased stability and expression of NRF2. Therefore, USP8, as a deubiquitinating enzyme, represents a potential therapeutic target for pancreatic cancer [123]. Recent studies have demonstrated that targeting deubiquitinases can effectively reduce the post-translational levels of NRF2 and YAP proteins, thereby decreasing the growth and chemoresistance of pancreatic cancer cells [124]. In animal experiments, researchers have found that the inactivation of Ubiquitin-Specific Peptidase 25(USP25) can alleviate oxidative liver injury induced by acetaminophen (APAP) overdose in male mice and reduce mortality caused by lethal doses of APAP. The mechanism primarily involves the direct binding of USP25 to Keap1, preventing its ubiquitination and degradation [125]. Lastly, in treating other diseases, modulating NRF2 deubiquitination in skin macrophages affects pain perception[126] (Table 4).

**Table 4:** Targeted drugs for NRF2 in ubiquitination and deubiquitination.

Drug	Function	References
Metformin+CDDP	Downregulate NRF2	[98]
MSU38225	Downregulate NRF2	[99]
DHT	Downregulate NRF2	[102]
SIT	Downregulate NRF2	[103]
Brucein D	Downregulate NRF2	[104]
Baicalin	Downregulate NRF2	[107]
Metformin	Downregulate NRF2	[116]
DUB3 inhibitor	Downregulate NRF2	[117]
RSL3	Inhibition of the NRF2-GSH pathway	[122]
GEM	NRF2 upregulation	[123]

### Other PTMs of NRF2

In addition to the above-mentioned PTMs, NRF2 undergoes a variety of other PTMs that also play important roles in regulating its stability, activity, and cellular localization.

#### SUMOylation

SUMOylation, or small ubiquitin-like modifier modification, involves the covalent attachment of SUMO proteins to specific lysine residues on target proteins. This is a reversible post-translational modification that regulates various cellular processes [127]. NRF2 SUMOylation is mediated by a sequential enzymatic process involving the SUMO-activating enzyme E1, the SUMO-conjugating enzyme E2, and the SUMO ligase E3 [128]. SUMOylation regulates the transcriptional activity of NRF2, affects its interaction with other proteins and intracellular localization, and protects NRF2 from ubiquitination-mediated degradation [129]. SUMOylation contributes to cellular resistance to oxidative stress and regulates antioxidant gene expression by enhancing NRF2 stability and activity [130]. In studies of Hepatocellular Carcinoma (HCC), SUMOylation enhances the ability of NRF2 to scavenge ROS and upregulate PHGDH, promoting de novo serine synthesis. Notably, serine deprivation enhanced NRF2 SUMOylation, which contributed to sustained HCC growth. Consequently, NRF2 SUMOylation plays a key role in promoting signaling pathways that support HCC tumorigenesis, both under normal conditions and in response to metabolic stress [131]. In the investigation of KRAS/LKB1/KEAP1 mutant lung adenocarcinoma cells, mild oxidative stress was observed to decrease NRF2 SUMOylation, which in turn enhanced the migration and invasion of these cells [132]. ERK5 is a two-kinase transcription factor, and phosphorylation of ERK5 S496 directly inhibits NRF2 protein activity through SUMOylation modification at site 518, an inhibitory effect that can lead to inflammation and mitochondrial dysfunction in macrophages [133].

#### Glycosylation

Glycosylation is a biochemical process in which sugar molecules are attached to proteins, lipids, or other organic molecules. Glycosylation is a type of post-translational modification of proteins that has important implications for protein structure, stability, function, and intracellular localization [134]. Common types of glycosylation include N-linked glycosylation and O-linked glycosylation [135–137]. Glycosylation plays a crucial role in cellular stress responses and signaling is closely linked to the regulation of NRF2. NRF2 activity is modulated by fructosamine-3-kinase (FN3K), which facilitates protein deglycosylation. In the absence of FN3K, NRF2 undergoes extensive glycosylation, resulting in decreased stability and transcriptional activation. The development of HCC triggered by Myelocytomatosis and Keap1 inactivation is dependent on FN3K in vivo. Treatment with N-acetylcysteine partially rescued the tumor-promoting effects driven by NRF2 in FN3K-deficient models, underscoring the importance of NRF2-mediated redox homeostasis [138–140]. Specific studies on the direct relationship between glycosylation and NRF2 are still limited, and more studies may further reveal the specific mechanisms and roles of glycosylation in NRF2 regulation.

#### PARylation

Upon sensing DNA damage (e.g., single-strand breaks), PARPase (especially PARP1) is activated and rapidly accumulates at the site of damage. The activated PARPase utilizes NAD^+^ as a substrate to transfer ADP-ribose units one by one to the target protein, forming poly ADP-ribose chains. These chains can be attached to PARPase itself (self-PARylation) or other proteins involved in DNA repair, gene expression regulation, and cellular stress response, a process known as poly-ADP-ribosylation (PARylation) [141]. PARylation can be importantly linked to NRF2 by modulating its stability and function [142], as well as by regulating DNA repair and antioxidant stress responses. Studies have shown that the PARP inhibitor Olaparib prevents chronic hypoxia/reoxygenation-induced retinal damage by modulating NRF2 [143]. Research on this post-translational modification pathway is also limited, with much more potential waiting to be explored.

## Epigenetic regulation of NRF2

Epigenetic modifications, such as DNA methylation, histone modifications, and interactions with non-coding RNAs, are critical in regulating NRF2 activity. These modifications are often reversible and can control the expression, activity, and stability of the NRF2 gene, as well as its interactions with other regulatory elements within the cell. By modulating the chromatin structure and accessibility of the NRF2 promoter, epigenetic modifications determine how effectively NRF2 can be activated in response to various cellular stresses. This regulation enables cells and organisms to swiftly and adaptively respond to fluctuating environmental conditions. In recent years, the influence of epigenetic modifications on NRF2 has gained increasing attention, highlighting their significance in the broader context of cellular stress responses and disease progression [144–146].

### DNA methylation

DNA methylation is an epigenetic process where a methyl (Me) group is added to specific cytosine residues within the DNA. By modifying gene expression without changing the underlying DNA sequence, this process predominantly occurs in CpG islands, regions where cytosine is positioned next to guanine [147]. The DNA methylation process of NRF2 regulates its gene expression by adding Me groups to the CpG islands in its promoter region. DNA methyltransferases (DNMTs) specifically facilitate the transfer of methyl groups from S-adenosylmethionine to cytosine residues, resulting in the formation of 5-methylcytosine. Methylation of the CpG islands in the NRF2 gene promoter typically suppresses NRF2 transcriptional activity, leading to reduced expression levels and impacting the cell’s antioxidant functions [148].

Recent research has demonstrated that plant-based dietary compounds can modulate NRF2 expression via epigenetic modifications. By inhibiting epigenetic enzymes like DNMTs, compounds such as curcumin and apigenin reduce CpG hypermethylation in the NRF2 promoter, leading to elevated NRF2 levels and offering potential therapeutic benefits for diseases associated with oxidative stress [35, 149]. Sulforaphane (SFN) can reduce CpG methylation in the NRF2 promoter region induced by Ang II and promote histone H3 acetylation in this region, thereby enhancing NRF2 expression. These epigenetic modifications contribute to the long-term cardioprotective effects of SFN [150]. Additionally, SFN combined with 5-aza-2’-deoxycytidine and trichostatin A significantly reduced the CpG methylation levels in the NRF2 promoter region and inhibited the protein expression of DNA methyltransferase 1 (DNMT1), thereby promoting NRF2 expression, which may play a preventive role in colon cancer [151]. In Chronic Obstructive Pulmonary Disease (COPD), hypermethylation of the NRF2 promoter leads to decreased NRF2 expression in the lungs of patients, resulting in the inhibition of the NRF2-GPX4 axis and subsequent induction of ferroptosis. This process is closely linked to the onset and progression of COPD [152]. In studies of non-alcoholic fatty liver disease, RSV was found to decrease the expression of lipogenic genes and triglyceride levels by attenuating NRF2 promoter methylation induced by high-fat diets and high glucose treatments, while also modulating NRF2 signaling pathways [153].

### DNA demethylation

DNA demethylation involves the removal of methyl groups from cytosine residues in DNA, typically mediated by demethylases such as TET1, TET2, and TET3. This process begins with the oxidation of 5-methylcytosine to 5-hydroxymethylcytosine, followed by further oxidation to 5-formylcytosine and 5-carboxylcytosine, with the oxidized intermediates eventually removed through the base excision repair mechanism, restoring the DNA to an unmethylated state. Specifically, demethylation of the NRF2 gene promoter region can activate NRF2 expression, enhancing the binding of transcription factors and regulatory proteins to the promoter, thereby increasing the transcription and protein expression of NRF2. As a key transcription factor, NRF2 regulates antioxidant and detoxifying enzymes, bolstering the cell’s ability to resist oxidative stress, toxins, and other harmful stimuli, which can improve disease progression and prognosis [154, 155].

Lignans have demonstrated anticancer properties in colon cancer cells by promoting apoptosis. This effect is mediated through the upregulation of NRF2 transcription, which is facilitated by the demethylation of its promoter region [156]. Tanshinone IIA (TIIA) enhances NRF2 binding and transcriptional activation at the promoters of target transporter proteins BSEP and NTCP through TET2-mediated demethylation of NRF2. This suggests that NRF2 plays a crucial role in rifampicin-induced liver injury, which can be mitigated by TIIA [157]. Nano-SiO2 can trigger malignant transformation and induce global DNA hypomethylation in human bronchial epithelial cells. Under these conditions, the increased demethylation levels of Cytosine-phosphate-Guanine islands in the NRF2 promoter region can lead to elevated expression of the NRF2 gene, thereby inhibiting the carcinogenic effects induced by Nano-SiO2 [154]. Redox imbalance is a key pathogenic mechanism in melanoma and non-melanoma skin cancers, with activation of the NRF2-ARE pathway acting as an intrinsic defense against oxidative stress. Delphinidin, a potent and abundant anthocyanin found in berries, can activate the NRF2 promoter by promoting its demethylation. This activation of the NRF2-ARE pathway positions delphinidin as a potential chemopreventive agent for skin cancer [158]. Geraniol, a compound in the anthocyanin family, decreases DNA methylation in the NRF2 promoter region of mouse skin epidermal JB6 (JB6 P+) cells and enhances the expression of NRF2 downstream target genes. Its potential anticancer effects may be linked to the activation of the NRF2-ARE signaling pathway and its associated cytoprotective properties [155]. Additionally, the study found that the demethylation of the NRF2 promoter region plays a promoting role in the development of CRC. Compared to precancerous polyps and normal tissues, the demethylation of the NRF2 promoter is significantly increased in tumor tissues, leading to NRF2 overexpression and promoting tumor progression. This finding suggests that targeting NRF2 could be a potential strategy for the prevention or treatment of CRC [159]. Pterostilbene (PTS) decreases the expression of HDAC and DNMT, leading to the demethylation of the NRF2 promoter and subsequent reactivation of NRF2. This reactivation triggers the transcription of the antioxidant gene SOD2, which helps counteract ROS-induced stress and supports wound healing under diabetic conditions. PTS’s ability to modulate these epigenetic mechanisms underscores its potential therapeutic benefits [160].

In summary, the DNA methylation and DNA demethylation modification of NRF2 plays a critical role in regulating its expression and function. Understanding this process is essential for elucidating the role of NRF2 in cellular stress response and disease protection. Future studies should delve deeper into the mechanisms of NRF2 methylation and its potential therapeutic applications (Table 5).

**Table 5:** Targeted drugs for NRF2 in DNA methylation and demethylation.

Drug	Function	References
Curcumin	NRF2 upregulation	[149]
Apigenin	NRF2 upregulation	[149]
SFN	NRF2 upregulation	[150, 151]
RSV	NRF2 upregulation	[153]
Lignans	NRF2 upregulation	[156]
TIIA	NRF2 upregulation	[157]
Nano-SiO2	NRF2 upregulation	[154]
Delphinidin	NRF2 upregulation	[158]
Geraniol	NRF2 upregulation	[155]
PTS	NRF2 upregulation	[160]

### Chromatin remodeling

Chromatin remodeling refers to the dynamic process of regulating gene expression by altering chromatin structure. This process involves the repositioning or restructuring of nucleosomes, thereby changing the accessibility of DNA to transcription factors and regulatory proteins, which in turn affects the expression of NRF2 target genes. This mechanism plays a critical role in the cellular response to oxidative stress and the regulation of antioxidant gene expression, and it may also be significant in disease progression [148].

In lung cancer, the loss of BRG1 or BRM components of the SWI/SNF chromatin remodeling complex activates the NRF2 signaling pathway, particularly increasing the expression of NRF2 target genes HMOX1 and GSTM4. This alteration is associated with poor patient prognosis [161]. DPF2 is an essential component of the hematopoiesis-specific BAF (SWI/SNF) chromatin remodeling complex. The loss of DPF2 impairs the function of NRF2-regulated enhancers, resulting in reduced expression of antioxidant and anti-inflammatory genes. This results in impaired macrophage polarization and excessive proliferation of hematopoietic stem cells. Pharmacological activation of NRF2 can mitigate the inflammation and lethality caused by DPF2 deficiency [162]. In NSCLC, persistently activated NRF2 creates distinct enhancers at gene loci that are not typically regulated by NRF2 under normal physiological conditions. This enhancer remodeling drives tumor-initiating activity and malignant progression by establishing the NRF2-NOTCH3 regulatory axis [163].

### Non-coding RNA regulation

Non-coding RNA regulation refers to the mechanisms by which non-coding RNAs (ncRNAs) modulate gene expression and cellular functions. Non-coding RNAs do not encode proteins but influence gene activity through various pathways. The principal types include microRNAs (miRNAs), long non-coding RNAs (lncRNAs), small interfering RNAs (siRNAs), and circular RNAs (circRNAs). These non-coding RNAs regulate NRF2 expression and activity through different mechanisms, affecting cellular responses to oxidative stress, inflammation, and other physiological processes [164, 165].

In heart failure, non-coding RNAs, particularly miRNAs encapsulated in extracellular vesicles (EV), influence the redox balance in the myocardium and brain by regulating the NRF2 signaling pathway. As a key antioxidant transcription factor, NRF2 activity decreases during heart failure, while miRNAs modulate NRF2 and its associated antioxidant proteins through EV, thereby regulating oxidative stress [166]. Cardiovascular risk factors induce ROS production, triggering oxidative stress and leading to disease. The long non-coding RNA MALAT1 activates the NRF2 transcription factor, enhancing the expression of antioxidant genes such as HO1, NQO1, and GCLC, which helps protect cells from oxidative stress-induced damage [167]. The long non-coding RNA MALAT1 suppresses NRF2 expression through epigenetic mechanisms, promoting inflammasome activation and ROS production in Parkinson’s disease (PD) mouse and microglial cell models, thereby exacerbating neuroinflammation [168]. Long non-coding RNA PVT1 enhances doxorubicin resistance in breast cancer cells by inhibiting the binding of Keap1 to NRF2, thereby preventing NRF2 protein degradation. Furthermore, the Keap1/NRF2/ARE signaling pathway plays a critical role in tumor cell drug resistance, and PVT1 may further promote the development of resistance by regulating this pathway [169]. Notably, targeted inhibition of NRF2 with Brusatol counteracts this effect, effectively suppressing NRF2 activity and inducing apoptosis in breast cancer cells both in vitro and in vivo [170]. In colorectal cancer research, upregulation of the long non-coding RNA MIR4435-2HG suppresses the expression of NRF2 and HO-1, thereby promoting the development of cisplatin resistance [171]. Long non-coding RNA TUG1 enhances cisplatin resistance in esophageal squamous cell carcinoma cells by directly binding to and increasing the levels of NRF2 protein [172].

## Summary of clinical trials involving NRF2

In the previous sections, we have explored the significant role of NRF2 in various diseases. To gain a more comprehensive understanding of the potential clinical applications of NRF2, the following table summarizes relevant clinical trial information. These trials encompass studies of both NRF2 activators and inhibitors in diverse application contexts, including treatments for different diseases, research strategies, and clinical trial phases. This data will aid us in gaining deeper insights into the actual effects and research progress of NRF2 in therapeutic settings, highlighting its important value and potential directions for future research and clinical applications (Table 6).

**Table 6:** Summary of clinical trials involving NRF2.

Disease	Medicine	Strategy	Phase	NCT
HNSCC	Pyrimethamine	Downregulate NRF2 pathway activity	I	NCT05678348
PD	Sulphoraphane	Activation of the Keap1/NRF2/ARE pathway	II	NCT05084365
Doxorubicin-associated cardiac dysfunction	Sulforaphane	Activation of the NRF2 signaling pathway	[44]	NCT03934905
CKD	Sulforaphane	Activation of the NRF2 signaling pathway	II	NCT05797506
Acute Coronary Syndrome	Ezetimibe	Activation of the NRF2 signaling pathway	–	NCT04701242
Primary Hypercholesterolemia or Mixed Dyslipidemia	Ezetimibe	Activation of the NRF2 signaling pathway	–	NCT04579367
Schizophrenia	Luteolin	Inhibit the expression of NRF2	–	NCT05204407
Adrenomyeloneuropathy	Dimethyl Fumarate	Activation of the NRF2 signaling pathway	[47]	NCT06513533
Multiple Sclerosis	Dimethyl fumarate	Activation of the NRF2 signaling pathway	IV	NCT05658484
Gulf War Illness	Resveratrol	Activation of the NRF2 signaling pathway	–	NCT05377242
Advanced or metastatic HR-positive/HER-2-Negative breast cancer	Fulvestrant	Activation of the NRF2 signaling pathway	I	NCT06172322
Ataxia	Omaveloxolone	Activation of the NRF2 signaling pathway	I	NCT06054893
Rare Chronic Kidney Diseases	Bardoxolone Methyl	Activation of the NRF2 signaling pathway	II	NCT03366337
Pre Diabetes	Eriocitrin	Activation of the NRF2 signaling pathway	–	NCT03928249
Sickle cell disease	Isoquercitrin	Activation of the NRF2/ARE Pathway	II	NCT04514510
Refractory Low-risk MDS	Retinoic Acid	Activate retinoic acid receptors to inhibit NRF2	I/II	NCT06020833
Coronary Artery Disease	Curcumin	Modification of Keap1 cysteine stabilizes NRF2 expression	–	NCT04458116
Acute Lymphoblastic Leukemia in Children	Curcumin	Modification of Keap1 cysteine stabilizes NRF2 expression	II	NCT05045443
Diabetic Patients With Atherosclerotic Cardiovascular Risk	Curcumin	Modification of Keap1 cysteine stabilizes NRF2 expression	II	NCT05753436
Subjective Tinnitus	Curcumin	Modification of Keap1 cysteine stabilizes NRF2 expression	–	NCT04800107
Colistin-induced Nephrotoxicity	Curcumin	Modification of Keap1 cysteine stabilizes NRF2 expression	III	NCT05613361
Postoperative Adhesion of Bilateral Vocal Cords	Curcumin	Modification of Keap1 cysteine stabilizes NRF2 expression	I/II	NCT05688488
Crohn’s Disease	Curcumin	Modification of Keap1 cysteine stabilizes NRF2 expression	II	NCT04713631
Childhood Lupus Nephritis	Curcumin	Modification of Keap1 cysteine stabilizes NRF2 expression	II	NCT05714670
Obsessive Compulsive Disorder	Pyridoxine	Activation of the NRF2/HO-1 Pathway	–	NCT06244121
OC, NSCLC, EC	Brusatol	Specifically inhibiting NRF2 expression	[173]	–

## Conclusion

In summary, NRF2, a key regulator of the cellular antioxidant stress response, is influenced by various PTMs and epigenetic modifications. These modifications, including phosphorylation, acetylation, ubiquitination, SUMOylation, glycosylation, and PARylation, not only regulate NRF2’s activity but also affect its interactions with other proteins, stability, and its role in critical cellular signaling pathways. Additionally, epigenetic modifications, including DNA methylation, histone modifications, and interactions with non-coding RNAs, are essential for regulating NRF2 expression and function. The complex interactions and coordination between these known and unknown PTMs and epigenetic modifications collectively determine the functional status of NRF2.

Importantly, pharmacological modulation of NRF2 has emerged as a promising therapeutic strategy. For instance, NRF2 inhibitors, such as Brusatol and Brucein D, promote ubiquitination and proteasomal degradation of NRF2, enhancing chemosensitivity in cancers like AML, endometrial cancer, and pancreatic ductal adenocarcinoma. Metformin, alone or combined with cisplatin, downregulates NRF2 to counteract chemoresistance, while MSU38225, DHT, and Baicalin suppress NRF2 activity to disrupt redox adaptation in tumors. Conversely, NRF2 activators like SFN, RSV, and curcumin enhance NRF2-mediated antioxidant defenses, offering cytoprotection in degenerative and inflammatory conditions. Compounds such as propofol and tBHQ upregulate NRF2 via PKC or epigenetic pathways, supporting cellular stress adaptation.

An in-depth investigation of these mechanisms will not only help to elucidate the complex regulatory network of NRF2 in cellular protective mechanisms but also provide new targets and strategies for the treatment of related diseases. Future research should focus on exploring the interactions between different modifications and their dynamic changes under physiological and pathological conditions. This will contribute to a comprehensive understanding of the NRF2 regulatory network and provide a theoretical foundation for developing novel therapeutic strategies. By understanding the roles of these modifications, epigenetic regulations, and pharmacological agents in cellular responses to external stresses and disease progression, we can more effectively utilize NRF2 as a therapeutic target, offering new hope for future health management and disease treatment. The dual role of NRF2, as a protector in normal cells and a survival driver in cancer, underscores the need for context-specific modulation, balancing antioxidant support with targeted inhibition to optimize therapeutic outcomes.
